# Diagnosis and treatment protocol for COVID‐19 patients (Tentative 10th Version)

**DOI:** 10.1002/hcs2.36

**Published:** 2023-02-23

**Authors:** Released by National Health Commission of People's Republic of China & National Administration of Traditional Chinese Medicine on January 5, 2023

**Keywords:** clinical guideline, COVID‐19, diagnosis, discharge criteria, treatment

## Abstract

To further improve the diagnosis and treatment of COVID‐19, the National Health Commission of People's Republic of China and the National Administration of Traditional Chinese Medicine convened a group of experts to revise the relevant content of the Diagnosis and Treatment Protocol for COVID‐19 Patients (Trial Version 9) and developed the Diagnosis and Treatment Protocol for COVID‐19 Patients (Trial Version 10), summarizing the etiological characteristics, epidemiological characteristics, prevention, clinical features, diagnosis, clinical classification, population with high risk of severe/critical illnesses, early warning predictors for severe/critical illnesses, differential diagnosis, case identification and reporting, treatment, nursing, control of nosocomial infection in medical institutions, and discharge criteria for inpatients.

**Abbreviations**
ACE‐2: angiotensin converting enzyme 2
CRP: C‐reactive protein
CRRT: continuous renal replacement therapy
ECMO: extracorporeal membrane oxygenation
ESR: erythrocyte sedimentation rate
FiO_2_
: fraction of inspired oxygen
HFNC: high‐flow oxygen therapy
IL: interleukin
IVIG: intravenous immunoglobulin
MIS‐C: multiple system inflammatory syndromes in children
NIV: noninvasive ventilation
PaO_2_
: partial pressure of oxygen
qF‐PCR: quantitative fluorescence polymerase chain reaction
RBD: receptor‐binding domain
RR: respiratory rate
SpO_2_
: pulse oxygen saturation
TCM: traditional Chinese medicine
VAV: veno‐arterial‐venous
VOC: variants of concern
VV: venous‐venous
WHO: World Health Organization

In order to further improve the diagnosis and treatment of COVID‐19, China National Health Commission and the National Administration of Traditional Chinese Medicine convened a group of experts to revise the relevant content of the *Diagnosis and Treatment Protocol for COVID‐19 Patients (Tentative 9th Version)* [1] and developed the *Diagnosis and Treatment Protocol for COVID‐19 Patients (Tentative 10th Version)*.

## 1 ETIOLOGICAL CHARACTERISTICS

The 2019‐nCoV (also as SARS‐CoV‐2) belongs to the beta genus of coronaviruses. It has an envelope, round or oval particles, and a diameter of 60–140 nm. It has four structural proteins of nucleoprotein (N), envelope protein (E), matrix protein (M), and spike protein (S). The 2019‐nCoV has a single‐stranded, positive‐sense RNA (+RNA) genome of 29.9 kb. The genome of 2019‐nCoV is typically organized in the order of 5′‐leader‐UTR‐replicase (ORF1a/ORF1b)‐S‐ORF3a‐ORF3b‐E‐M‐ORF6‐ORF7a‐ORF7b‐ORF8‐N‐ORF9a‐ORF9b‐ORF10‐3′. The N protein wraps the RNA genome to form a nucleocapsid, which is the core of the virus particle and is surrounded by a lipid bilayer that contains the S, the M, and the N proteins. In the respiratory tract, the S protein of the 2019‐nCoV enters the host cell by recognizing and binding to angiotensin converting enzyme 2 (ACE‐2) through its receptor‐binding domain (RBD). The 2019‐nCoV undergoes frequent genetic mutations during its spread among the population. When multiple subtypes or subvariants of the 2019‐nCoV infect the human body at the same time, genetic recombination will also occur to produce recombinant strains; Certain genetic mutations or recombinations may affect the biological characteristics of the 2019‐nCoV. For example, mutations of amino acids in the spike protein will strengthen the binding affinity of the 2019‐nCoV and ACE‐2 and enhance the virus's ability of cell replication and transmission. Some amino acid mutations in the spike protein also increase the 2019‐nCoV's ability to evade vaccinations and reduce cross‐protection among variants, leading to breakthrough infection and reinfection.

As of the end of 2022, there are five “variants of concern” (VOC) defined by the World Health Organization (WHO), namely Alpha (B.1.1.7), Beta (B.1.351), Gamma (P.1), Delta (B.1.617.2), and Omicron (B.1.1.529). The Omicron variant appeared in the population in November 2021. Compared with other VOC variants such as Delta, it is more transmissible with increased immune escape. In early 2022, the Omicron variant quickly replaced the Delta variant to become the dominant variant globally. Up to now, 709 subclades have evolved from five Omicron clades (BA.1, BA.2, BA.3, BA.4, and BA.5), of which 72 are recombined.

As the global spread of the 2019‐nCoV continues, new subvariants of Omicron will continue to emerge. BA.5.2 was a predominant Omicron variant that circulated globally for several months. Since October 2022, BF.7, BQ.1, and BQ.1.1, as well as the sublineage recombinant XBB, which are more immune evasive and transmissible, have spread rapidly and replaced BA.5.2 as the predominant strains in some countries and regions.

Available evidence at home and abroad shows that the Omicron variant demonstrates weakened lung pathogenicity, and the clinical presentation has changed from mainly pneumonia to mainly upper respiratory tract infections. The Omicron variant does not impact the he 2019‐nCoV detection capability of RT‐PCR assays diagnostic, but it may reduce the neutralizing effect of some monoclonal antibody drugs.

Coronavirus is sensitive to ultraviolet rays, organic solvents (ether, 75% ethanol, peracetic acid, chloroform, etc.) and chlorine‐containing disinfectants. Seventy‐five percentage ethanol and chlorine‐containing disinfectants are commonly used to inactivate the virus in clinics and laboratories, while chlorhexidine cannot.

## 2 EPIDEMIOLOGICAL CHARACTERISTICS

### 2.1 Source of infection

The source of infection is mainly patients infected with the 2019‐nCoV as well as asymptomatic carriers. Patients are infectious during the incubation period and are highly infectious within 3 days after the onset of the disease.

### 2.2 Route of transmission

a. The main route of transmission of 2019‐nCov is respiratory droplet transmission and close contact transmission.
b. The virus may spread through aerosols in a relatively closed environment.
c. Contact with items contaminated by the virus can also cause infection.

### 2.3 Susceptible population

Everyone is susceptible to 2019‐nCoV. Infection or vaccination can acquire certain immunity.

The elderly and patients with severe underlying diseases have a higher rate of severe illness and fatality after infection than the general population, and such rates can be reduced after vaccination.

## 3 PREVENTION

### 3.1 Vaccination

Vaccination is an effective means of reducing the incidence of critical illness and death, resulting in a reduction in the 2019‐nCoV infection and morbidity. People who meet the requirements for vaccination and booster vaccination should be vaccinated in a timely manner.

### 3.2 General precautions

People should maintain good personal hygiene and a clean environment surrounding them, with balanced nutrition, proper exercise, adequate rest, and no excessive fatigue. They should wash hands frequently, wear masks, keep the “one meter line” and use public chopsticks. Covering mouths and noses when sneezing or coughing and keeping the room ventilated well are encouraged for personal protection.

## 4 CLINICAL FEATURES

### 4.1 Clinical manifestations

The incubation period is mostly 2 to 4 days. The main symptoms are dry throat, sore throat, cough, and fever. Most patients have low to moderate fever, but in some cases patients can have high fever, which does not exceed 3 days. Some patients may present with muscle aches, decreased or lost sense of smell and/or taste, nasal congestion, runny nose, diarrhea, and conjunctivitis. In a small number of patients, the disease continues to progress with ongoing fever and pneumonia‐related manifestations. Severe patients often develop dyspnea and/or hypoxemia within 5 to 7 days after the onset. Critically ill cases can quickly progress to acute respiratory distress syndrome, septic shock, irreversible metabolic acidosis, coagulation dysfunction, and multiple organ failure. A very small number of patients may also have central nervous system involvement.

The clinical manifestations of infection in children are similar to those in adults, with high fever being relatively common; some cases may have atypical symptoms, such as vomiting, diarrhea, and other gastrointestinal symptoms or only poor response and shortness of breath; a few children may develop acute laryngitis or laryngotracheitis, such as hoarseness, wheezing, and pulmonary rales, but rarely severe respiratory distress; a few children may develop febrile convulsions, and a very small number of children may develop encephalitis, meningitis, encephalopathy, and even acute necrotizing encephalopathy, acute disseminated encephalomyelitis, Guillain‐Barré syndrome, and other life‐threatening neurological complications. Multiple system inflammatory syndromes (MIS‐C) may also occur, mainly manifesting as fever with rash, non‐purulent conjunctivitis, mucosal inflammation, hypotension or shock, coagulopathy, acute gastrointestinal symptoms, and encephalopathic manifestations such as convulsions and cerebral. Once this occurs, the condition can deteriorate sharply in a short period of time.

Most patients have a good prognosis, with a few patients being critically ill. Most of the critically ill patients are more common in the elderly, those with chronic underlying diseases, women in late pregnancy and perinatal period, or obese people.

### 4.2 Laboratory diagnosis

#### 4.2.1 General testing

In the early stage of the disease, peripheral white blood cell counts are normal or decreased as well as the lymphocyte count. Some patients may have increased liver enzymes, lactate dehydrogenase, muscle enzymes, myoglobin, troponin, and ferritin. C‐reactive protein (CRP) and erythrocyte sedimentation rate (ESR) is elevated and normal procalcitonin level in most patients. Severe and critically ill patients can show increased D‐dimer level, a progressive decrease in lymphocyte counts, and an increased level of inflammatory factors.

#### 4.2.2 Etiology and serological examination

a. Nucleic acid test: nucleic acid amplification tests can be used to detect the 2019‐nCoV's nucleic acid in respiratory specimens (nasopharyngeal swabs, throat swabs, sputum, and tracheal aspirates) or other specimens. Quantitative fluorescence PCR (qF‐PCR) is the most commonly used method.
b. Antigen detection: the detection of viral antigen in respiratory specimens by colloidal gold method and immunofluorescence method is fast and its sensitivity is positively correlated with the viral load of the infected person; a positive viral antigen test supports the diagnosis, but a negative test cannot deny the diagnosis.
c. Cell culture isolation: the 2019‐nCoV can be isolated by cell culture from respiratory samples, fecal samples, and so forth.
d. Serological examination: the 2019‐nCoV‐specific IgM antibody and IgG antibody are positive, and the positive rate is low within 1 week of onset. When IgG antibody is increased by four times or more in the convalescent phase compared to the acute phase, a retrospective diagnosis can be conducted.

### 4.3 Chest imaging

In the early stage of patients complicated with pneumonia, multiple small patchy shadows and interstitial changes are seen, especially in the periphery of the lung. Then it develops into multiple ground glass shadows and infiltration shadows in both lungs. Severe cases can show lung consolidation, but pleural effusion is rare.

## 5 DIAGNOSIS

### 5.1 Diagnostic principles

Diagnosis should be made on the basis of a comprehensive analysis of epidemiological history, clinical manifestations, and laboratory tests. A positive nucleic acid test for the new coronavirus is the primary criterion for diagnosis.

### 5.2 Diagnostic criteria

a. Have clinical manifestations associated with 2019‐nCoV infection;
b. Have one or more of the following pathogenic and serological findings;
c. Positive nucleic acid test for 2019‐nCoV;
d. Positive antigen test for 2019‐nCoV;
e. Positive for antigen isolated by cell culture;
f. IgG antibodies are increased by four times or more in the convalescent phase compared to the acute phase.

## 6 CLINICAL CLASSIFICATION

### 6.1 Mild

Upper respiratory tract infection as the main manifestation, such as dry throat, sore throat, cough, fever, and so forth.

### 6.2 Moderate

Persistent high fever >3 days or (and) cough and shortness of breath, but respiratory rate (RR) <30 breaths/min and oxygen saturation >93% on air inhalation at rest. Characteristic pneumonic manifestations of 2019‐nCoV infection can be seen on imaging.

### 6.3 Severe

Adults meeting any one of the following, which cannot be explained by other reasons other than the 2019‐nCoV infection:

a. Shortness of breath, RR ≥ 30 times/min;
b. In the resting state, the pulse oxygen saturation(SpO_2_） is ≤93% while breathing ambient air;
c. Arterial partial pressure of oxygen (PaO_2_)/the fraction of inspired oxygen (FiO_2_) ≤300 mmHg (1 mmHg = 0.133 kPa);

In areas with high altitude (more than 1000 m above sea level), PaO_2_/FiO_2_ should be adjusted according to the following formula: PaO_2_/FiO_2_×[760/atmospheric pressure (mmHg)].

d. The clinical symptoms are progressively worse, and lung imaging shows that the lesion has progressed significantly >50% within 24 to 48 h.

Children meeting any of the following:

a. High fever lasting more than 3 days; Shortness of breath (<2‐month‐old, RR ≥ 60 beats/min; 2–12 months old, RR ≥ 50 beats/min; 1 to 5‐year‐old, RR≥40 beats/min; >5‐year‐old, RR ≥ 30 times/min), the influence of fever and crying excluded;
b. In the resting state, the pulse oxygen saturation is ≤93% while breathing ambient air;
c. Respiratory distress (nostril flapping, three concave signs, wheezing or stridor);
d. Impaired consciousness and convulsions;
e. Refusal to feed or feeding difficulties with signs of dehydration.

### 6.4 Critical

Meet any one of the following conditions:

a. Respiratory failure and mechanical ventilation;
b. Shock;
c. ICU admission due to other organ failures.

## 7 POPULATION WITH HIGH RISK OF SEVERE/CRITICAL ILLNESSES

a. Older than 65 years, especially those who have not received the full course of 2019‐nCoVvaccines;
b. Have the comorbidities such as cardio‐cerebrovascular diseases (including hypertension), chronic lung diseases, diabetes, chronic liver or kidney disease, and tumors, and patients on maintenance dialysis;
c. Immune function deficiency (AIDS patients, long‐term use of corticosteroids or other immunosuppressive drugs that lead to immune suppression);
d. Obesity (body mass index ≥30);
e. Late pregnancy and perinatal women;
f. Heavy smokers.

## 8 EARLY WARNING PREDICTORS FOR SEVERE/CRITICAL ILLNESSES

### 8.1 Adults

The following indicators should be alert to the deterioration

a. Hypoxemia or progressive exacerbation of respiratory distress;
b. Deterioration of tissue oxygenation (eg., mixed venous oxygen saturation) or progressive increase in lactate;
c. Progressive decrease of peripheral blood lymphocyte count or an increase in inflammatory markers such as IL‐6, CRP, and ferritin;
d. Significant increase of D‐dimer and coagulation dysfunction;
e. Chest imaging showed an obvious progression of pneumonia.

### 8.2 Children

a. Increased breathing rate;
b. Poor mental reactivity, lethargy, convulsions;
c. Decreased peripheral blood lymphocyte count and (or) thrombocytopenia;
d. Hypoglycemia/hyperglycemia and (or) elevated lactic acid;
e. Markedly elevated inflammatory factors such as PCT, CRP, ferritin;
f. Markedly increased AST, ALT, CK;
g. Significantly elevated coagulation‐related indicators such as D‐dimer;
h. Head imaging with changes such as cerebral edema or chest imaging showing significant progression of lung lesions;
i. Have underlying diseases.

## 9 DIFFERENTIAL DIAGNOSIS

a. Manifestations of 2019‐nCoV infection must be differentiated from upper respiratory tract infections caused by other viruses.
b. Manifestations of 2019‐nCoV infection needs to be distinguished from other known viral types of pneumonia including influenza virus, adenovirus, respiratory syncytial virus, and mycoplasma pneumoniae infection.
c. Differentiation from noninfectious diseases such as vasculitis, dermatomyositis, and organizing pneumonia.
d. Children with rashes and mucosal damage should be differentiated from Kawasaki disease.

## 10 CASE IDENTIFICATION AND REPORTING

Medical institutions at all levels and of all types should report directly on the national network for infectious diseases in accordance with the law.

## 11 TREATMENT

### 11.1 General treatment

a. Isolated and treated according to the requirements of respiratory infectious diseases. Ensure adequate energy and nutrition intake, pay attention to the balance of water and electrolyte to maintain homeostasis. For those with high fever, physical cooling and antipyretic drugs should be applied. Give cough and expectorant drugs to those with severe cough and sputum.
b. Monitor vital signs, especially oxygen saturation at rest and after activity, in high‐risk patients with severe illness. Also monitor the indicators related to the underlying disease.
c. Monitor blood routine, urine routine, CRP, biochemical variables (liver enzymes, myocardial enzymes, kidney function, etc.), blood coagulation function, arterial blood gas analysis, chest imaging, and so forth.
d. Supply effective oxygen therapy including nasal cannula, mask oxygen, and nasal high flow oxygen therapy according to patient condition.
e. Antibacterial treatment: Avoid inappropriate use of antibacterial drugs, especially the combined use of broad‐spectrum antibacterial drugs.
f. Those with underlying diseases should be treated accordingly.

### 11.2 Antiviral treatment

#### 11.2.1 Nirmatrelvir tablets/ritonavir tablets (co‐packaged)

Indicators are mild‐to‐moderate adult COVID‐19 patients within 5 days of disease onset and high‐risk factors of severe disease. Usage: 300 mg of nirmatrelvir with 100 mg of ritonavir, given twice daily for 5 days. The drug instructions should be read carefully before use, and it should not be used in combination with drugs such as meperidine and ranolazine, which are highly dependent on CYP3A for clearance and whose plasma concentration increases can lead to serious and/or life‐threatening adverse reactions. They should be used during pregnancy only if the potential benefit to the mother outweighs the potential risk to the fetus. Use during lactation is not recommended. Nematavir should be reduced by half in people with moderate renal impairment and should not be used in people with severe hepatic or renal impairment.

#### 11.2.2 Azvudine tablets

It can be used for the treatment of adult patients with moderate SARS‐CoV‐2 infection. Usage: the tablet should be swallowed whole and taken without food. Five milligrams should be taken every 24 h for a maximum of 14 days. Read the instructions carefully before use and be aware of interactions with other drugs and adverse reactions. It is not recommended for use during pregnancy and lactation, and should be used with caution in patients with moderate to severe hepatic or renal impairment.

#### 11.2.3 Molnupiravir capsule

Indicators are mild‐to‐moderate adult COVID‐19 patients within 5 days of disease onset and high‐risk factors of severe disease. Usage: 800 mg should be taken orally every 12 h for 5 days. It is not recommended for use during pregnancy and lactation.

#### 11.2.4 Monoclonal antibodies

Ambavirumab/Romisevirumab Injection. Used in combination for adults and adolescents (12–17 years old, weight ≥40 kg) with mild to moderate COVID‐19 and high‐risk factors of severe disease. Usage: two drugs are administered at a dose of 1000 mg respectively. After being diluted with 100 mL of normal saline, the two drugs are administered by intravenous sequential infusion at a rate of not higher than 4 mL/min, and 100 mL of normal saline is used to flush the tube in between. Clinically monitor patients during infusion and at least 1 h after infusion.

#### 11.2.5 Intravenous immunoglobulin

Intravenous immunoglobulin therapy for COVID‐19 can be used among high‐risk rapidly deteriorating patients with high viral load in the early stage of the disease. The recommended dosage for mild cases is 100 mg/kg, 200 mg/kg for the moderate case, and 400 mg/kg for the severe illness. The second infusion can be administered the next day, depending on the improvement of the patient's condition, with the total number not exceeding five.

#### 11.2.6 Convalescent plasma

It can be used in patients with high‐risk factors, a high viral load, and rapid disease progression in the early stage of the disease. The dosage is 200 to 500 mL (4 to 5 mL/kg), and whether to administer should be determined according to the individual patient's situation and viral load.

#### 11.2.7 Other drugs approved by the National Medical Products Administration for the treatment of COVID‐19

### 11.3 Immunotherapy

#### 11.3.1 Glucocorticoid

For patients with progressive deterioration of oxygenation, rapid imaging progression, and excessive inflammatory responses, glucocorticoids can be used for a short duration (3 to 5 days, and no more than 10 days). The recommended dosage is 5 mg/day for dexamethasone and is 40 mg/day for methylprednisolone. Larger doses and long‐term use of glucocorticoids should be avoided to reduce side effects.

#### 11.3.2 Interleukin‐6 (IL‐6) inhibitor: Tocilizumab

Eligible for patients with severe and critical patients with elevated IL‐6 levels. Specific usage: The first dose is 4–8 mg/kg, with the recommended dose of 400 mg, 0.9% saline to dilute to 100 mL, and the infusion time of more than 1 h. If the first dose is not effective, the same dose can be applied 12 h after the first dose. The maximum number of administrations is two and the maximum single dose does not exceed 800 mg. Allergic reactions must be monitored. Contraindicated in active infections, such as tuberculosis.

### 11.4 Anticoagulation

For the moderate patient with high‐risk factors and rapid disease progression, severe and critically ill patients, therapeutic doses of low molecular weight heparin or unfractionated heparin should be used when there is no contraindication. Treatment should be provided in case of thromboembolism according to the guidelines.

### 11.5 Prone position

The moderate patients with high risk and rapid disease progression, severe and critically ill patients should be given standardized prone position therapy for longer than 12 h per day.

### 11.6 Psychological intervention

Patients often have tension and anxiety. Psychological counseling should be strengthened and supplemented with drug treatment if necessary.

### 11.7 Supportive treatment of severe and critical cases

#### 11.7.1 Treatment principle

Prevent and treat complications, treat underlying diseases, prevent secondary infections, and provide timely organ function support based on the above management.

#### 11.7.2 Respiratory support

##### Nasal cannula or face mask oxygen therapy

Patients with PaO_2_/FiO_2_ lower than 300 mmHg should receive oxygen therapy immediately. Patients who receive oxygen supplement via a nasal cannula or face mask oxygen therapy must be closely monitored for 1–2 h. If respiratory distress and/or hypoxemia does not improve, nasal high‐flow oxygen therapy (HFNC) or noninvasive ventilation (NIV) should be used.

##### Nasal high‐flow oxygen therapy or noninvasive ventilation

Patients with PaO_2_/FiO_2_ less than 200 mmHg should receive HFNC or NIV. We recommend that patients who receive HFNC or NIV should receive awake prone position ventilation for at least 12 h if there is no contraindication.

Some patients have a high risk of HFNC or NIV treatment failure. Any patients receiving HFNO and NIV should be monitored closely. If there is no improvement in hypoxemia or the frequency of breathing or patients have excessive tidal volume or excessive inspiratory effort, especially after the prone position treatment within 1–2 h, invasive mechanical ventilation should be performed immediately.

##### Invasive mechanical ventilation

If the PaO_2_/FiO_2_ is less than 150 mmHg, especially in patients with significantly enhanced inspiratory effort, endotracheal intubation and invasive mechanical ventilation should be considered. However, given the atypical clinical manifestations of hypoxemia in severe and critically ill patients, PaO_2_/FiO_2_ should be evaluated in real‐time in combination with clinical manifestations and organ functions other than itself for endotracheal intubation and invasive mechanical ventilation. It is worth noting that the delay of endotracheal intubation may cause greater harm.

Early and appropriate invasive mechanical ventilation is important for critically ill patients. Lung protective mechanical ventilation strategies must be implemented. Lung recruitment manipulation can be performed in patients with moderate to severe acute respiratory distress syndrome with a FiO_2_ higher than 50%. Repeatedly implement pulmonary recruitment manipulation should consider lung recrutability. It should be noted that some patients with COVID‐19 have poor recrutiablity lung and high PEEP should be avoided.

##### Airway management

We recommend using an active heating humidifier and loop heating guide wire if possible for airway humidification. Closed suction and tracheostomy suction if necessary are recommended. Airway clearance therapy such as vibration expectoration, high‐frequency thoracic oscillation, and postural drainage is recommended. Passive and active activities should be performed as soon as possible to promote sputum drainage and pulmonary rehabilitation in the case of stable oxygenation and hemodynamics.

##### Extracorporeal membrane oxygenation (ECMO)

ECMO indications: patients who receive the optimal mechanical ventilation conditions (FiO_2_≥80%, tidal volume of 6 mL/kg ideal body weight, PEEP≥5 cmH_2_O, and no contraindications) and prone ventilation meet any one of the following criteria should be considered to implement ECMO as soon as possible:

a. PaO_2_/FiO_2_<50 mmHg over 3 h;
b. PaO_2_/FiO_2_<80 mmHg over 6 h;
c. Arterial blood pH<7.25 and PaCO_2_>60 mmHg for more than 6 h, and respiratory rate (RR)>35 times/min;
d. RR >35 times/min, arterial blood pH <7.2 and the plateau pressure >30 cmH_2_O;

Critically ill patients who meet the ECMO indications should receive ECMO treatment as soon as possible if there is no contraindication.

ECMO mode selection: Venous‐venous ECMO (VV‐ECMO) is the most commonly used for respiratory support; Venous‐arterial ECMO (VA‐ECMO) is used for patients who need both respiratory and circulatory support. veno‐arterial‐venous EMCO (VAV‐ECMO) should be considered when the differential hypoxia is developing in patients receiving VA‐ECMO. Lung protective ventilation strategies must be performed after ECMO treatment. Recommended initial settings: tidal volume <4–6 mL/kg ideal body weight, plateau pressure ≤25 cmH_2_O, driving pressure <15 cmH_2_O, PEEP 5–15 cmH_2_O, breathing rate 4–10 times/min, FiO_2_<50%. We recommend prone position ventilation for patients whose oxygenation is difficult to maintain, or with a strong inspiratory effort, obvious consolidation of the gravity‐dependent areas of the lungs, or active drainage of airway secretions

#### 11.7.3 Circulation support

Critically ill patients can be complicated with shock. On the basis of adequate fluid resuscitation, vasoactive drugs should be used reasonably. Blood pressure, heart rate, and urine output changes, as well as lactic acid and base excess should be closely monitored. Invasive hemodynamic monitoring should be used if necessary.

#### 11.7.4 Acute kidney injury and renal replacement therapy

Critically ill patients can be complicated with acute kidney injury. Some factors that induced AKI such as drugs and low perfusion should be considered. Maintain water, electrolyte, and acid–base balance while treating the cause of AKI. Indications for continuous renal replacement therapy (CRRT) include: (1) hyperkalemia; (2) severe acidosis; (3) pulmonary edema or excessive fluid overload with ineffective diuretics.

#### 11.7.5 Treatment of special conditions in children

##### Acute laryngitis or laryngotracheitis

First, the degree of upper airway obstruction and hypoxia should be assessed, and oxygen should be administered if there is hypoxia. At the same time, the air should be kept moist to avoid children's irritability and crying. Glucocorticoids is the first choice for mild cases, who can also take orally single‐dose dexamethasone (0.15−0.6 mg/kg, maximum dose 16 mg) or prednisolone (1 mg/kg). Dexamethasone (0.6 mg/kg, maximum dose 16 mg) is the first choice for moderate and severe cases to take orally, and intravenous or intramuscular injection can be administered if the patient is unable to take orally; 2 mg of budesonide can also be given via a nebulizer. For severe airway obstruction, tracheal intubation or tracheotomy and mechanical ventilation should be performed to maintain airway patency. In case of emergency, Inhalation of L‐epinephrine via a nebulizer can quickly relieve the symptoms of upper airway obstruction, and the recommended dose is 0.5 mL/kg (maximum 5 mL) for 15 min each time. If the symptoms are not relieved, the inhalation can be repeated after 15–20 min.

##### Lung wheezes

bronchodilators and glucocorticoids for aerosol inhalation can be used for patients based on comprehensive treatment; Salbutamol, Ipratropium bromide, and budesonide are also commonly used; for those with thick sputum, N‐acetylcysteine can be administered via a nebulizer.

##### Neurological complications such as encephalitis and encephalopathy

Body temperature should be controlled, and mannitol should be given to reduce intracranial pressure along with anticonvulsant therapy; tracheal intubation and mechanical ventilation are required for those with rapid disease progression; for patients with severe encephalopathy, especially acute necrotizing encephalopathy, methylprednisolone (20 to 30 mg/kg) should be given every 24 h for 3 days as early as possible, followed by gradual dose reduction depending on the disease; Intravenous immunoglobulin (IVIG) was administered intravenously at a total of 2 g/kg in 1 or 2 days. Plasma exchange, tocilizumab, or cocktail therapy that improves mitochondrial metabolism (vitamin B1, vitamin B6, levocarnitine, etc.) may also be used as appropriate. Treatment principles for encephalitis, meningitis, Guillain Barré syndrome are the same as for related disorders caused by other etiologies.

##### Children's multisystem inflammatory syndrome

The treatment principle is anti‐inflammatory treatment, correcting shock and coagulation dysfunction, and organ function support. Intravenous immunoglobulin (IVIG, 2 g/kg) and methylprednisolone (1–2 mg/kg/day) are the first choice for patients. When the condition does not improve or worsens, methylprednisolone (10−30 mg/kg/day) can be given intravenously, or the patient can use Infliximab (5−10 mg/kg) or tocilizumab.

#### 11.7.6 Severe or critical cases with pregnancy

Multiple‐discipline consultation should be sought for risk assessment. The pregnancy should be terminated if necessary. Cesarean section is the first choice.

#### 11.7.7 Nutritional support

Nutritional risk assessment should be strengthened. Enteral nutrition with a energy of 25−30 kcal/kg/day and protein >1.2 g/kg/day should be performed. Parenteral nutrition can be added if necessary. Intestinal microecological regulator can be used to maintain intestinal microecological balance and prevent secondary bacterial infection.

### 11.8 Traditional Chinese medicine (TCM) therapy

This disease belongs to plague in TCM, caused by epidemic pathogenic factors. According to the different local climate characteristics and individual states of illness and physical conditions, the following treatment Protocol may vary. The use of over‐pharmacopoeia doses should be directed by a physician. For nonkey populations with early‐stage COVID‐19 infection, home treatment can be carried out by using traditional Chinese medicine and Traditional Chinese medicine prescriptions stated in Guidelines for Home‐Based Traditional Chinese Medicine Intervention for Patients with COVID‐19 Infection and Notice on the Full Application of Traditional Chinese Medicine Decoction in the Treatment of COVID‐19 Infection at the Primary Level in Urban and Rural Areas.

#### 11.8.1 Treatment

##### Qingfei Paidu decoction

Scope of application: It is suitable for mild, moderate, severe, and critically ill patients, and can be used as per patients' conditions and medication requirements.

Prescription composition: Ma Huang (Ephedrae Herba) 9 g, Zhi Gan Cao (Glycyrrhizae Radix) 6 g, Xing Ren (Armeniacae Semen) 9 g, Sheng Shi Gao (Gypsum fibrosum) (decocted first) 15–30 g, Gui Zhi (Cinnamomi Ramulus) 9 g, Ze Xie (Alismatis Rhizoma) 9 g, Zhu Ling (Polyporus) 9 g, Bai Zhu (Atractylodis macrocephalae Rhizoma) 9 g, Fu Ling (Poria) 15 g, Chai Hu (Bupleuri Radix) 16 g, Huang Qin (Scutellariae Radix) 6 g, Jiang Ban Xia (Pinellinae Rhizoma Praeparatum) 9 g, Sheng Jiang (Zingiberis Rhizoma recens) 9 g, Zi Wan (Asteris Radix) 9 g, Kuan Dong Hua (Farfarae Flos) 9 g, She Gan (Belamcandae Rhizoma) 9 g, Xi Xin (Asari Radix et Rhizoma) 6 g, Shan Yao (Dioscoreae Rhizoma) 12 g, Zhi Shi (Aurantii Fructus immaturus) 6 g, Chen Pi (Citri reticulatae Pericarpium) 6 g, Guang Huo Xiang (Pogostemonis Herba) 9 g.

Suggested use: Traditional Chinese medicine decoction pieces for decocting in water. One dose daily with half of the dose taken in the morning and half in the evening (40 min after meal) with warm water. Three days make a course of treatment. If the patient has special conditions or other underlying diseases, the prescription of the second course of treatment can be modified based on the actual situation and the medicine should be discontinued when the symptoms disappear.

Recommended Chinese patent medicine: Qingfei Paidu granules.

##### Mild cases

a. Syndrome of superficies tightened by toxin
  Clinical manifestations: fever and headache with no sweat, sore body, itchy throat, cough or dry and sore throat, few phlegm, stuffy nose and muddy mucus. The tongue is red, the coating is thin white or yellow, and the pulse is floating.
  Prescription composition: Ge Gen (Puerariae Lobatae Radix) 15 g, Jin Jie (Schizonepeta Tenuifolia) 10 g, Chai Hu (Bupleuri Radix) 15 g, Huang Qin (Scutellariae Radix) 15 g, Bo He (Herba Menthae) 10 g, Gui Zhi (Cinnamomi Ramulus) 10 g, Bai Shao (Cynanchum otophyllum Schneid) 10 g, Jin Yin Hua (Lonicerae Japonicae Flos) 15 g, Jie Geng (Platycodonis Radix) 15 g, Zhi Qiao (Fructus Aurantii) 10 g, Qian Hu (Peucedani Radix) 15 g, Chuan Xiong (Ligusticum Chuanxiong Hort) 10 g, Bai Zhi (Radix Angelicae dahuricae) 10 g, Gan Cao (Glycyrrhizae Radix) 10 g.
  Suggested use: one dose daily, boiled with 100–200 mL water, finish orally the dose(s) in 2–4 times across the day. The following prescriptions are taken in the same way (if the patient presents special manifestations, please follow the doctor's instructions).
b. Cold‐dampness and stagnation lung syndrome
  Clinical manifestations: fever, fatigue, sore body, cough, expectoration, nausea, sticky stools. The tongue body looks pale and puffy, the coating is white thick rot or white greasy, and the pulse is soggy or slippery.
  Recommended prescription: epidemic due to cold‐dampness formula
  Prescription composition: Ma Huang (Ephedrae Herba) 6 g, Sheng Shi Gao (Gypsum fibrosum) 15 g, Ku Xing Ren (Armeniacae Semen) 9 g, Qiang Huo (Notopterygii Rhizoma seu Radix) 15 g, Ting Li Zi (Lepidii/Descurainiae Semen) 15 g, Mian Ma Guan Zhong (Cyrtomii Rhizoma) 9 g, Di Long (Pheretima) 15 g, Xu Chang Qing (Cynanchi paniculati Radix) 15 g, Huo Xiang (Pogostemonis Herba) 15 g, Pei Lan (Eupatorii Herba) 9 g, Cang Zhu (Atractylodis Rhizoma) 15 g, Fu Ling (Poria) 45 g, Bai Zhu (Atractylodis macrocephalae Rhizoma) 30 g, Jiao Mai Ya (Hordei Fructus germinatus) 9 g, Jiao Shan Zha (Crataegi Fructus) 9 g, Jiao Shen Qu (Massa medicata fermentata) 9 g, Hou Po (Magnoliae officinalis Cortex) 15 g, Jiao Bing Lang (Arecae Semen) 9 g, Cao Guo (Tsaoko Fructus) 9 g, Sheng Jiang (Zingiberis Rhizoma recens) 15 g.
c. Dampness and heat‐accumulation lung syndrome
  Clinical manifestations: fever, sore body, dry and sore throat, dry mouth without desire of drinking much water; cough with little sputum, or accompanied by chest tightness, loss of appetite, diarrhea or sticky stool. The tongue is puffy and reddish, and the coating is white, thick, and greasy or yellow, and the pulse is slippery or soggy.
  Recommended prescription: Bing Lang (Arecae Semen) 10 g, Cao Guo (Tsaoko Fructus) 10 g, Hou Po (Magnoliae officinalis Cortex) 10 g, Zhi Mu (Anemarrhenae Rhizoma) 10 g, Huang Qin (Scutellariae Radix) 10 g, Chai Hu (Bupleuri Radix) 10 g, Chi Shao (Paeoniae Radix Rubra) 10 g, Lian Qiao (Forsythiae Fructus) 15 g, Qing Hao (Artemisiae annuae Herba) (added later) 10 g, Cang Zhu (Atractylodis Rhizoma) 10 g, Da Qing Ye (Isatidis Folium) 10 g, Gan Cao (Glycyrrhizae Radix) 5 g.
  Recommended Chinese patent medicine: Huoxiang Zhengqi capsules (pills, liquid, or oral solution), Shufeng Jiedu capsules (granules), Qingfei Paidu granules, Huashi Baidu granules, Xuanfei Baidu granules, Sanhan Huashi granules, Jinhua Qinggan granules, Lianhua Qingwen capsules (granules).
  Recommended acupuncture points for acupuncture treatment: Hegu, Houxi, Yinlingquan, Taixi, Feishu, Pishu. Acupuncture method: select three acupoints each time. The acupuncture adopts the method of flat tonic and flat catharsis. The degree of Qi is obtained. Keep the needle for 30 min once a day. The methods for following acupuncture points are the same as this one.

##### Moderate cases

a. Dampness and stagnation lung syndrome
  Clinical manifestations: fever, cough, aversion to wind and cold, body aches, dry throat and sore throat, suffocation, bloating, and constipation. The tongue is dark red and fat; the coating is greasy or yellow and the pulse is slippery or stringy.
  Recommended prescription: lung‐diffusing and toxin‐resolving formula.
  Prescription composition: Ma Huang (Ephedrae Herba) 6 g, Ku Xing Ren (Armeniacae Semen) 15 g, Sheng Shi Gao (Gypsum fibrosum) 30 g, Sheng Yi Ren (Coicis Semen) 30 g, Cang Shu (Atractylodis Rhizoma) 10 g, Guang Huo Xiang (Pogostemonis Herba) 15 g, Qing Hao (Artemisiae annuae Herba) 12 g, Hu Zhang (Polygoni cuspidati Rhizoma) 20 g, Ma Bian Cao (Verbenae Herba) 30 g, Lu Gen (Phragmitis Rhizoma) 30 g, Ting Li Zi (Lepidii/Descurainiae Semen) 15 g, Hua Ju Hong (Citri grandis Exocarpium rubrum) 15 g, Gan Cao (Glycyrrhizae Radix) 10 g.
b. Cold‐dampness lung syndrome
  Clinical manifestations: low fever, submerged fever or absence of fever, dry cough, scanty sputum, fatigue, chest tightness, stuffy and full sensation in the stomach, or nausea, loose stool. The tongue is pale or red, and the coating is white or greasy, and the pulse is soggy.
  Recommended prescription: Cang Shu (Atractylodis Rhizoma) 15 g, Chen Pi (Citri reticulatae Pericarpium) 10 g, Hou Po (Magnoliae officinalis Cortex) 10 g, Guang Huo Xiang (Pogostemonis Herba) 10 g, Cao Guo (Tsaoko Fructus) 6 g, Ma Huang (Ephedrae Herba) 6 g, Qiang Huo (Notopterygii Rhizoma seu Radix) 10 g, Sheng Jiang (Zingiberis Rhizoma recens) 10 g, Bing Lang (Arecae Semen) 10 g.
c. Plague poison and dryness syndrome
  Clinical manifestations: fever, cough, dry and sore throat, constipation, light red tongue, less fluid, thin white or dry coating, and tight pulse.
  Recommended prescription: lung‐diffusing, dryness moistening, and toxin‐resolving formula.
  Prescription composition: Ma Huang (Ephedrae Herba) 6 g, Ku Xing Ren (Armeniacae Semen) 15 g, Chai Hu (Radix Bupleuri) 12 g, Sha Seng (Radix Ginseng) 15 g, Mai Dong (*Ophiopogon japonicus*) 15 g, Xuan Seng (Radix Scrophulariae) 15 g, Bai Zhi (Radix Angelicae dahuricae) 10 g, Qiang Huo (notopterygium) 15 g, Sheng Ma (Cimicifuga) 8 g, Sang Ye (Mori Folium) 15 g, Huang Cen (Scutellaria baicalensis) 10 g, Sang Bai Pi (mulberry bark) 15 g, Sheng Shi Gao (Gypsum fibrosum) 20 g.
  Recommended Chinese patent medicine: Jinhua Qinggan granules, Lianhua Qingwen capsules (granules), Qingfei Paidu granules, Huashi Baidu granules, Xuanfei Baidu granules, Sanhan Huashi granules.
  Recommended acupuncture points for acupuncture treatment: Neiguan, Kongzui, Quchi, Qihai, Yinlingquan, Zhongwan.

##### Severe cases

a. Plague poison and lung‐closing syndrome
  Clinical manifestations: fever, shortness of breath, chest tightness, cough, yellow and sticky phlegm, or blood in sputum, wheezing, bitterness and stickiness in the mouth, poor stool, and short urination. The tongue is red; the coating is yellow greasy and the pulse is slippery.
  Recommended prescription: dampness‐removing and toxin‐resolving formula.
  Prescription composition: Ma Huang (Ephedrae Herba) 6 g, Ku Xing Ren (Armeniacae Semen) 9 g, Sheng Shi Gao (Gypsum fibrosum) (decocted first) 15 g, Gan Cao (Glycyrrhizae Radix) 3 g, Guang Huo Xiang (Pogostemonis Herba) 10 g, Hou Po (Magnoliae officinalis Cortex) 10 g, Cang Zhu (Atractylodis Rhizoma) 15 g, Cao Guo (Tsaoko Fructus) 10 g, Fa Ban Xia (Pinellinae Rhizoma Praeparatum) 9 g, Fu Ling (Poria) 15 g, Sheng Da Huang (Rhei Radix et Rhizoma) (added later) 5 g, Huang Qi (Astragali Radix) 10 g, Ting Li Zi (Lepidii/Descurainiae Semen) 10 g, Chi Shao (Paeoniae Radix rubra) 10 g.
b. Blazing of both qi and ying syndrome
  Clinical manifestations: Hot fever, thirst, shortness of breath, delirium and unconsciousness, or spotted rash, or hemoptysis, or convulsions in the limbs. The tongue is crimson with little or no coating. The pulse is deep, fine and rapid, or floating, large and rapid.
  Recommended prescription: Sheng Shi Gao (Gypsum fibrosum) (decocted first) 30–60 g, Zhi Mu (Anemarrhenae Rhizoma) 30 g, Sheng Di (Rehmanniae Radix) 30–60 g, Shui Niu Jiao (Bubali Cornu) (decocted first) 30 g, Chi Shao (Paeoniae Radix rubra) 30 g, Xuan Shen (Scrophulariae Radix) 30 g, Lian Qiao (Forsythiae Fructus) 15 g, Dan Pi (Moutan Cortex) 15 g, Huang Lian (Coptidis Rhizoma) 6 g, Zhu Ye (Phyllostachys nigrae Folium) 12 g, Ting Li Zi (Lepidii/Descurainiae Semen) 15 g, Gan Cao (Glycyrrhizae Radix) 6 g.
  Suggested use: 1 dose per day, decoction, boiled with 100–200 mL water, finish the dose(s) in 2–4 times across the day, orally or nasally.
c. Poison and lung‐invading syndrome due to yang qi deficiency
  Clinical manifestations: Chest tightness, shortness of breath, pale complexion, cold limbs, fatigue, nausea, poor appetite, loose stools. The tongue body is pale, with less or white coating, and the pulse is deep and fine or weak.
  Recommended prescription: Qi‐strengthening and toxin‐resolving formula.
  Prescription composition: Fu Pian (Aconiti Radix lateralis praeparata) 10 g, Gan Jiang (Zingiberis Rhizoma) 15 g, Gan Cao (Glycyrrhizae Radix) 20 g, Jin Yin Hua (Lonicerae Japonicae Flos) 10 g, Zao Jiao Ci (Gleditsiae Spina) 10 g, Wu Zhi Mao Tao (Ficus hirta Vahl)/Huang Qi (Astragali Radix) 20 g, Guang Huo Xiang (Pogostemonis Herba) 10 g, Chen Pi (Citri reticulatae Pericarpium) 5 g.
  Suggested use: 1 dose per day, decoction, boiled with 100–200 mL water, finish the dose(s) in 2–4 times across the day, orally or nasally.
d. Recommended Chinese patent medicine: Please see “Recommended Chinese patent medicine for severe and critically ill cases.”
e. Recommended acupuncture points for acupuncture treatment: Dazhui, Feishu, Pishu, Taixi, Lieque, Taichong.

##### Critically ill cases

a. Internal blockage and external desertion syndrome
  Clinical manifestations: dyspnea, asthma, fainting, irritability, sweating, cold limbs, dark purple tongue, thick greasy or dry coating, and large floating pulse without root.
  Recommended prescription: Ren Shen (Ginseng Radix) 15 g, Hei Fu Pian (Aconiti Radix lateralis praeparata) (decocted first) 10 g, Shan Zhu Yu (Corni Fructus) 15 g, delivered with Suhexiang Pill or Angong Niuhuang Pill.
b. Recommended acupuncture points for acupuncture treatment: Taixi, Shanzhong, Guanyuan, Baihui, Zusanli, Suliao.
c. Recommended Chinese patent medicine for severe and critically ill cases: Qingfei Paidu granules, Huashi Baidu granules, Xiyanping injection, Xuebijing injection, Reduning injection, Tanreqing injection, Xingnaojing injection, Shenfu injection, Shengmai injection, Shenmai injection. Drugs with similar efficacy can be selected according to individual conditions, or can be used in combination according to clinical symptoms. Traditional Chinese medicine injection can be used in combination with TCM decoction.
d. Medication for severe and critically ill cases according to the patient's symptoms.

Patients with high fever can use Angong Niuhuang Pills (take 0.5 pill at a time, 2 to 4 times a day).

Patients with abdominal distension, constipation or poor stool (gastrointestinal dysfunction) can take Da Cheng Qi Tang, which consists of Sheng Da Huang (Rhei Radix et Rhizoma) 30 g, Mang Xiao (Natrii Sulfas) 30 g, Hou Po (Magnoliae officinalis Cortex) 15 g, and Zhi Shi (Aurantii Fructus Immaturus) 20 g, via enema. They can also take 5 to 30 g Sheng Da Huang (Rhei Radix et Rhizoma, decoction pieces or powder) by decocting or mixing it with water 2 to 4 times each day, until bowel movement is 1 to 3 times per day.

Patients with diarrhea or even watery stools can use Huoxiang Zhengqi capsules (softgels, pills, water, liquid).

Patients with chest tightness and shortness of breath (respiratory distress) can take a decoction of Gualou Xiebai Banxia decoction and Wuling Powder, which consists of Gua Lou (Trichosanthis Fructus) 30 g, Xie Bai (Allium macrostemon) 15 g, Fa Ban Xia (Pinellinae Rhizoma Praeparatum) 15 g, Fu Ling (Poria) 30 g, Zhu Ling (Polyporus umbellatus) 30 g, Ze Xie (Alisma Orientalis) 30 g, Gui Zhi (Cinnamomi Ramulus) 10 g, Bai Zhu (Atractylodis macrocephalae Rhizoma) 20 g, and Ting Li Zi (Lepidii/Descurainiae Semen) 15 g. (200 mL of thick decoction, orally or nasal feeding in 3 to 4 times).

For patients with coma, drowsiness and other disturbance of consciousness, Suhe Xiang Pill can be used orally or dissolved in water for nasal feeding (take 1 pill at a time, 1–2 times a day).

For patients with fatigue, shortness of breath, weakness, spontaneous sweating and poor appetite, 15 to 30 g of Xi Yang Shen (Panacis Quinquefolii Radix), Sun‐cured Ginseng, or Hong Shen (Radix Ginseng Rubra) can be decocted for oral feeding (200 mL of thick decoction, orally or nasal feeding in 3 to 4 times).

Patients with pale face, cold climbs, and aversion to wind can take a decoction of 10 g of Fu Pian (Aconiti Radix lateralis praeparata), 15 g of Gan Jiang (Zingiberis Rhizoma), and 15–30 g of Hong Shen (Radix Ginseng Rubra) (200 mL of thick decoction, orally or nasal feeding in 3 to 4 times).

Patients with dry months and lips, red tounge with pale coating can take a decoction of 20–30 g of Xi Yang Shen (Panacis Quinquefolii Radix), 15 g of Mai Dong (Ophiopogonis Radix), and 15 g of Xuan Shen (Radix Scrophulariae). (200 mL of thick decoction, orally or nasal feeding in 3 to 4 times).

Patients with profuse sweating and cold limbs (shock) can take a decoction of over 30 g of Fu Pian (Aconiti Radix lateralis praeparata) (decocted in advance for 2 h), 20g of Gan Jiang (Zingiberis Rhizoma), 30 g of Hong Shen (Radix Ginseng Rubra) and 30 g of Huang Qi (Astragali Radix) after using the prescription for the internal blockage and external desertion syndrome. (200 mL of thick decoction, orally or nasal feeding in 3 to 4 times).

Patients with edema of the face and limbs (cardiac insufficiency) can take a decoction of Wuling Powder, which consists of 30 g of Fu Ling (Poria), 30 g of Zhu Ling (Polyporus umbellatus), 30 g of Ze Xie (Alisma Orientalis), 10 g of Gui Zhi (Cinnamomi Ramulus), 20 g of Bai Zhu (Atractylodis macrocephalae Rhizoma), 30 g of Da Fu Pi (Arecae Pericarpium), 10 g of Qing Pi (Citri Reticulatae Pericarpium Viride), and 15 g of Ting Li Zi (Lepidii/Descurainiae Semen), after using the prescription for the internal blockage and external desertion syndrome. (200 mL of thick decoction, orally or nasal feeding in 3 to 4 times).

##### Convalescent period

a. Lung and spleen qi deficiency syndrome
  Clinical manifestations: shortness of breath, fatigue, anorexia, nausea, fullness, loose stool, and uneasiness. The tongue is pale and greasy.
  Recommended prescription: Fa Ban Xia (Pinellinae Rhizoma Praeparatum) 9 g, Chen Pi (Citri reticulatae Pericarpium) 10 g, Dang Shen (Codonopsis Radix) 15 g, Zhi Huang Qi (Astragali Radix) 30 g, Chao Bai Zhu (Atractylodis macrocephalae Rhizoma) 10 g, Fu Ling (Poria) 15 g, Huo Xiang (Pogostemonis Herba) 10 g, Sha Ren (AmomiFructus) (added later) 6 g, Gan Cao (Glycyrrhizae Radix) 6 g.
b. Deficiency of both qi and yin syndrome
  Clinical manifestations: fatigue, shortness of breath, dry mouth, thirst, palpitations, sweating, poor appetite, dry cough with scanty sputum, dry tongue, fine or weak pulse.
  Recommended prescription: Nan Sha Shen (Adenophorae Radix) 10 g, Bei Sha Shen (Glehniae Radix) 10 g, Mai Dong (Ophiopogonis Radix) 15 g, Xi Yang Shen (Panacis quinquefolii Radix) 6 g, Wu Wei Zi (Schisandrae Fructus) 6 g, Sheng Shi Gao (Gypsum fibrosum) 15 g, Dan Zhu Ye (Lophatheri Herba) 10 g, Sang Ye (Mori Folium) 10 g, Lu Gen (Phragmitis Rhizoma) 15 g, Dan Shen (Salviae miltiorrhizae Radix) 15 g, Sheng Gan Cao (Glycyrrhizae Radix) 6 g.
c. Coldness and stagnation lung syndrome
  Clinical manifestations: itching and coughing of the throat, or paroxysmal coughing, choking coughing, night coughing. The cough is aggravated by coldness and triggered by allergies, with white phlegm difficult to cough up. The tongue coating is white and greasy and the pulse is stringy and tense.
  Clinical manifestations: She Gan (Belamcandae Rhizoma) 9 g, Ma Huang (Ephedrae Herba) 6 g, Gan Jiang (Zingiberis Rhizoma) 15 g, Zi Wan (Asteris Radix) 30 g, Kuan Dong Hua (Farfarae Flos) 30 g, Wu Wei Zi (Schisandra Chinensis) 15 g, Fa Ban Xia (Pinellinae Rhizoma Praeparatum) 9 g, Qian Hu (Peucedani Radix) 15 g, Bai Bu (Stemonae Radix) 15 g, Su Zi (Perillae Fructus) 9 g, Ting Li Zi (Lepidii/Descurainiae Semen) 15 g, Chuan Bei Powder (Fritillaria cirrhosa) 3 g (taken with water).
d. Recommended acupuncture points for acupuncture treatment: Zusanli (moxibustion), Baihui, Taixi. Acupuncture points for indrect moxibustion: Dazhui, Feishu, Pishu, Kongzui (applied 40 min per day).

#### 11.8.2 Treatment for children

The TCM syndrome characteristics and core pathogenesis of children are basically the same as those of adults. The treatment is based on the adult TCM treatment scheme, with consideration of the clinical symptoms and the physiological characteristics of children.

##### Mild and moderate cases

a. Wind‐heat and dampness syndrome
  Clinical manifestations: fever, dry cough with little sputum, sore throat, stuffy and runny nose, irritability and crying, fatigue, poor appetite, red tongue with yellowish coating, floating pulse.
  Recommended prescription: Toxin‐resolving formula for children.
  Prescription composition: Ma Huang (Ephedrae Herba) 4 g, Sheng Shi Gao (Gypsum fibrosum) 12 g (decocted first), Ku Xing Ren (Armeniacae Semen) 5 g, Gan Cao (Glycyrrhizae Radix) 5 g, Guang Huo Xiang (Pogostemonis Herba) 9 g, Yi Yi Ren (Coicis Semen) 15 g, Lu Gen (Phragmitis Rhizoma) 10 g, Jie Geng (Platycodonis Radix) 6 g, Lian Qiao (Forsythiae Fructus) 9 g, Sheng Shan Zha (Crataegi Fructus) 10 g.
  Suggested use: Decocted into 100 mL per dose, 50ml for children ≤3‐year‐old, 100 mL for children 3–7 years old, 150–200 mL for children 7–14 years old; for those with high fever and acute illness, the dose can be increased as appropriate and can be taken in 2–3 times with warm water. For infants or children with difficulty in taking the medicine, it can be divided into several doses of 5 to 10 mL each with warm water.
b. Wind‐cold and dampness syndrome
  Clinical manifestations: fever and aversion to cold, headache and stuffy nose, cough, fatigue, nausea, loss of appetite, pale red tongue with white greasy coating, wet pulse.
  Recommended prescription: Maxing Yigan decoction and Shensu potion.
  Prescription composition: Ma Huang (Ephedrae Herba) 4 g, Yi Yi Ren (Coicis Semen) 10 g, Ku Xing Ren (Armeniacae Semen) 5 g, Su Ye (Perillae Folium) 6 g, Ge Gen (Puerariae Lobatae Radix) 10 g, Fu Ling (Poria) 10 g, Zhi Qiao (Fructus Aurantii) 6 g, Jie Geng (Platycodonis Radix) 6 g, Mu Xiang (Aucklandiae Radix) 5 g, Guang Huo Xiang (Pogostemonis Herba) 5 g, Chen Pi (Citri reticulatae Pericarpium) 3 g, Fang Feng (Saposhnikoviae Radix) 6 g, Tai Zi Shen (Pseudostellariae Radix) 5 g, Gan Cao (Glycyrrhizae Radix) 5 g.
  Suggested use: as stated in Section 11.8.2.1 (a).
c. Recommended Chinese patent medicine: Patients with persistent high fever, delirium, and a tendency to severe illness can be treated with Angong Niuhuang Pill, 1/6 pill for infants, 1/4 pill for children aged 3–6 years, and 1/3 to 1/2 pill for children aged 7–14 years, to be taken orally or intranasally once or twice a day in 5 mL of warm water. Patients with diarrhea and vomiting can take Huoxiang Zhenqi liquid (reduced dosage for patients aged under 5 years and normal dosage for patients aged over 5 years) or Huoxiang Zhenqi capsules (softgels, pills) (for patients aged over 5 years). Other Chinese herbal medicines with the effects of dispelling wind, relieving exterior symptoms, clearing heat, resolving dampness and toxins can be used as appropriate.
d. External treatment
  Tui Na therapy for children: push Tianmen 50–100 times, push Kangong 50–100 times, knead Taiyang 50–100 times, press Fengchi 5 times, clear Feijing 100 times, push the Bladder meridian 30 times on each side, push spine 50 times. If the main manifestation is fever, and the symptoms are wind‐heat and dampness, clear Feijing 100 times, push Sanguan 40 times and rub Liufu 120 times on the basis of the previous step; if the symptoms are wind‐cold and dampness, push Sanguan 90 times and rub Liufu 30 times on the basis of the previous step.
  Guasha (Scraping) Therapy: Apply oil on the front neck, chest and back, scrape for 5−10 min until red marks are seen.
  Acupuncture: For patients with sore throat, disinfect and puncture Shaoshang acupoint with a three‐edged needle to bleed; for patients with high fever that does not subside, disinfect and puncture the Erjian or Shuxuan acupuncture point gently with a three‐edged needle to bleed.

##### Severe and critically ill cases

Treatment for children with severe or critical illness can refer to the above‐mentioned treatment for adults with severe or critical illness. On the basis of syndrome differentiation, therapies that can facilitate catharsis, expel heat, resolve toxin, suppress convulsions and develop Qi should be highlighted. Angong Niuhuang Pills, Dushen Decoction can be used as appropriate for the treatment.

### 11.9 Early rehabilitation therapy

Attention should be given to early rehabilitation of patients with COVID‐19, and early rehabilitation training and intervention for respiratory and physical function, and psychological disorders should be actively implemented to restore physical fitness and immunity as much as possible.

## 12 NURSING

According to the patient's condition, nurses must clarify the key points of care and maintain proper basic care. In critically ill patients, close observation of patient's vital signs, state of consciousness and monitoring of blood oxygen saturation must be implemented. Critically ill patients must have 24h continuous ECG monitoring, measurements of the patient's heart rate, respiratory rate, blood pressure, and SpO_2_ every hour, as well as measuring and recording body temperature every 4 h. Venous access must be done correctly, and all conduits must be unobstructed and properly fixed. Bedridden patients must change their positions regularly to prevent pressure sores. Implementation of noninvasive mechanical ventilation, invasive mechanical ventilation, artificial airway, prone position ventilation, sedation and analgesia, and ECMO is done in accordance with nursing regulations. Special attention is needed for patients' oral care and fluid inflow and outflow management, as well as aspiration prevention in patients with invasive mechanical ventilation. Psychological assessment should be done for conscious patients and proper care provided.

## 13 CONTROL OF NOSOCOMIAL INFECTION IN MEDICAL INSTITUTIONS

a. Implement the pre‐screening triage system for emergency and outpatient care to ensure patient triage. Provide guidance on hand hygiene, respiratory hygiene and good cough habits. Patients with respiratory symptoms and accompanying personnel should wear medical surgical masks or medical protective masks.
b. Strengthen ward ventilation, and clean and disinfect object surfaces in consultation rooms, wards, offices, duty rooms and other areas.
c. Medical personnel should carry out appropriate personal protection according to the risk of exposure in accordance with the principle of standard precautions. They should wear medical surgical masks or medical protective masks during work, and strictly implement hand hygiene.
d. Dispose of medical waste according to requirements, and carry out final disinfection after patient transfer or discharge.

## 14 DISCHARGE CRITERIA FOR INPATIENTS

If the patient's condition has improved significantly with stable vital signs, the body temperature is normal for more than 24 h, the lung imaging shows significant improvement of acute exudative lesions, the patient can switch to oral therapy, and there are no complications that require further treatment, the patient can be considered for discharge.

The official notice of the release and the official Diagnosis and Treatment Protocol for COVID‐19 Patients (Trial Version 10) can be found at:

Official Notice, Letter No. 4 [2023] of the National Health Commission, http://www.nhc.gov.cn/xcs/zhengcwj/202301/32de5b2ff9bf4eaa88e75bdf7223a65a.shtml

The Official Protocol (Trial Version 10), http://www.nhc.gov.cn/xcs/zhengcwj/202301/32de5b2ff9bf4eaa88e75bdf7223a65a/files/02ec13aadff048ffae227593a6363ee8.pdf

## AUTHOR CONTRIBUTIONS

Group author–equal contribution.

## CONFLICT OF INTEREST STATEMENT

The author declares no conflict of interest.

## ETHICS STATEMENT

Not applicable.

## INFORMED CONSENT

Not applicable.

## Data Availability

Not applicable.
